# Extra-articular Endoscopic Treatment of Lateral Malleolar Tophaceous Gout: A Case Report

**DOI:** 10.7759/cureus.104589

**Published:** 2026-03-02

**Authors:** Masashi Koide, Satoshi Tateda, Taishi Murakami, Mika Abe

**Affiliations:** 1 Orthopedic Surgery, Japanese Red Cross Ishinomaki Hospital, Ishinomaki, JPN

**Keywords:** endoscopic surgery, extra-articular lesion, lateral malleolus, minimally invasive treatment, tophaceous gout

## Abstract

Gouty tophi are chronic manifestations of gout and are typically considered extra-articular lesions; however, periarticular extension around the ankle can complicate diagnosis and surgical decision-making. Open excision of lateral malleolar tophi is often associated with wound complications due to limited soft tissue coverage. A 56-year-old man presented with a progressively enlarging, painful mass over the lateral malleolus of the left ankle, causing difficulty wearing shoes. Imaging studies revealed a periarticular soft tissue mass with calcification and rice body-like inflammatory changes. Despite imaging findings suggestive of joint involvement, the lesion was clinically localized extra-articularly. Endoscopic excision was performed by directly inserting a trocar into the tophaceous mass to create a working tunnel, without introducing an arthroscope into the ankle joint. Extensive chalky deposits were successfully removed with minimal soft tissue disruption. Extra-articular endoscopic excision is a safe and effective minimally invasive option for treating lateral malleolar gouty tophi. This approach may obviate the need for intra-articular arthroscopy, even when periarticular inflammatory changes are present on imaging, and can provide favorable functional outcomes with reduced risk of wound complications.

## Introduction

Gout is a metabolic disorder characterized by the deposition of monosodium urate crystals and may progress to chronic tophaceous disease when serum uric acid levels remain uncontrolled [1,2]. Gouty tophi are generally regarded as extra-articular lesions; however, they may extend into adjacent joints, bursae, or tendon sheaths, particularly in the ankle and foot region [3-5]. Such periarticular involvement can complicate diagnosis and surgical decision-making.

Surgical intervention for gouty tophi is usually reserved for cases refractory to medical treatment or those causing functional impairment, pain, skin compromise, or difficulty with footwear [1,2]. Around the lateral malleolus, open excision is technically challenging because of thin soft tissue coverage and compromised local circulation, which increases the risk of wound complications.

Recent advances in endoscopic techniques have enabled minimally invasive treatment of gouty tophi, with reduced soft tissue disruption and improved wound healing. Endoscopic excision has been reported mainly for tophi of the first metatarsophalangeal joint and the olecranon region [6,7]. However, reports describing extra-articular endoscopic treatment of lateral malleolar gouty tophi are scarce.

In this report, we present a case of a large extra-articular gouty tophus located directly over the lateral malleolus, successfully treated using an endoscopic approach without intra-articular arthroscopy, and discuss its clinical significance. Medical management with urate-lowering therapy remains the cornerstone of gout treatment and is essential for preventing tophus progression and recurrence. However, large or symptomatic tophi may require surgical intervention when they cause mechanical symptoms, infection risk, or functional impairment.

## Case presentation

A 56-year-old man, a junior high school teacher, was referred to our orthopedic department with progressive swelling of the left lateral ankle for approximately three years. He complained of localized pain, recurrent redness, and increasing difficulty wearing shoes. The patient had a history of gout involving the first metatarsophalangeal joint and knee, with multiple acute flares over several years. Urate-lowering therapy had been prescribed previously but had been discontinued approximately five years before presentation. No other joints showed clinically apparent tophi at the time of evaluation, and he had no other medical treatment.

Physical examination revealed an approximately 5 cm in diameter, reddish soft tissue mass located directly over the lateral malleolus. Local warmth was present, but there was no skin ulceration or discharge. The patient was able to ambulate without claudication, and ankle range of motion was preserved.

**Figure 1:**
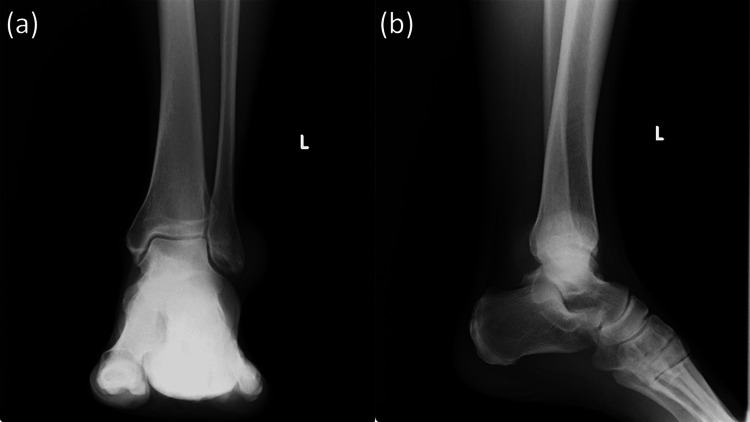
Preoperative ankle radiographs (a) Anteroposterior view and (b) lateral view of the left ankle show preserved joint space without evidence of bone erosion, osteoarthritis, or intra-articular calcification, suggesting the absence of advanced intra-articular involvement.

**Figure 2:**
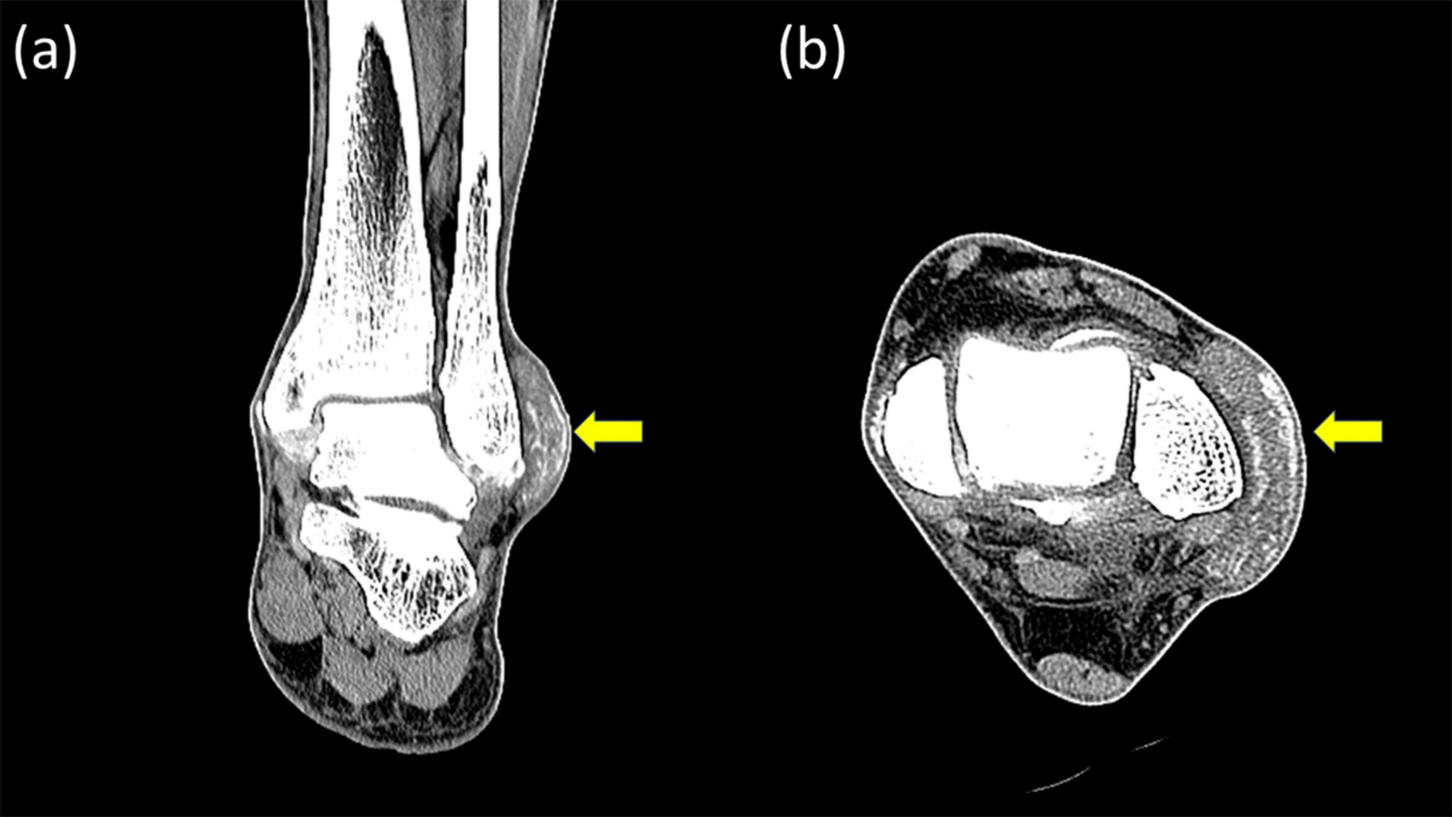
Preoperative computed tomography (a) Coronal and (b) axial computed tomography images demonstrate a nodular extra-articular soft tissue mass located directly over the lateral malleolus (arrow). Focal calcified deposits are observed along the surrounding ligamentous structures, including both lateral and medial ligaments. No bone erosion or definite intra-articular extension is identified.

Laboratory investigations demonstrated hyperuricemia, with a serum uric acid level of 9.4 mg/dL. C-reactive protein was within the normal range (0.05 mg/dL), and the white blood cell count was 6,200/μL (Table 1). Plain radiographs of the ankle showed no evidence of osteoarthritis or bony erosion (Figure 1). Computed tomography revealed a well-defined periarticular soft tissue mass with calcified deposits adjacent to the lateral malleolus, as well as calcifications along both the medial and lateral ankle ligaments (Figure 2), findings consistent with chronic urate deposition [3,4].

**Table 1:** Laboratory results CRP: C-reactive protein; WBC: white blood cell; UA: uric acid.

Parameter	Patient Value	Reference Range
CRP (mg/dL)	0.05	<0.30
WBC (/μL)	6200	3100-8400
UA (mg/dL)	9.4	2.0-7.0

**Figure 3:**
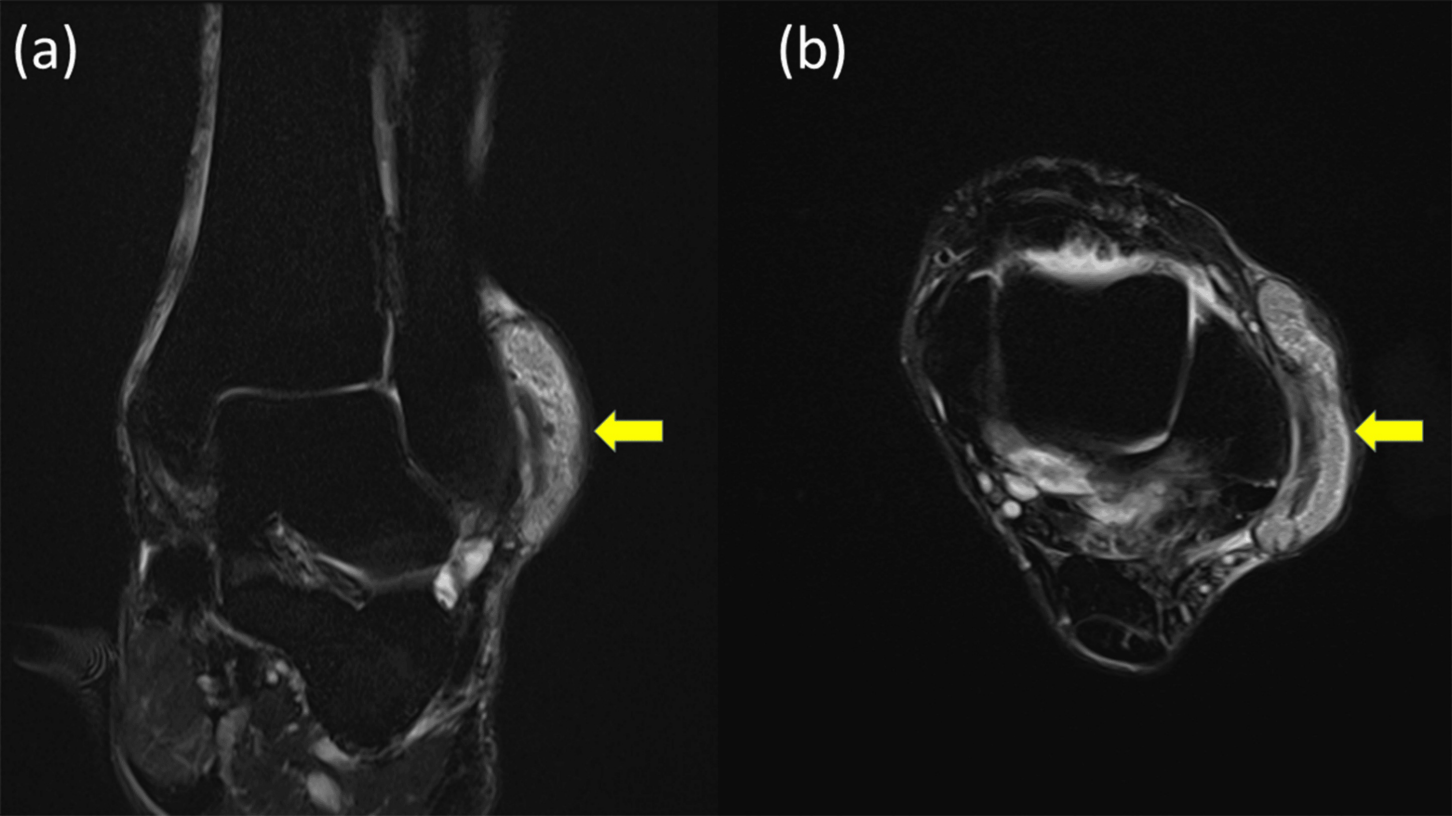
Preoperative magnetic resonance imaging (a) Coronal and (b) axial fat-suppressed T2-weighted magnetic resonance images demonstrate periarticular soft tissue inflammation with joint effusion. Multiple rice body-like structures are observed around the ankle, appearing as hyperintense nodular lesions within the extra-articular soft tissues (arrow). No definitive intra-articular tophaceous mass or articular cartilage destruction is identified.

Magnetic resonance imaging demonstrated joint effusion and marked distension of periarticular bursae containing numerous rice body-like structures (Figure 3). These lesions appeared isointense to muscle on T1-weighted images and slightly hyperintense on fat-suppressed T2-weighted images, findings that have been reported in chronic inflammatory conditions associated with gout [4].

Ultrasound-guided aspiration yielded white, chalky material consistent with tophaceous deposits. Polarized light microscopy was not performed preoperatively; however, the diagnosis of gout was subsequently confirmed by histopathological identification of monosodium urate crystal deposition. A corticosteroid injection was administered; however, symptoms failed to improve. Given the persistent functional impairment and failure of conservative management, surgical treatment was indicated [1,2]. Although MRI suggested possible communication with the ankle joint, clinical examination and CT demonstrated a well-localized extra-articular lesion without bone erosion or intra-articular extension. Based on this imaging-clinical correlation, the lesion was considered predominantly extra-articular, and isolated extra-articular endoscopic excision without ankle arthroscopy was planned.

**Figure 4:**
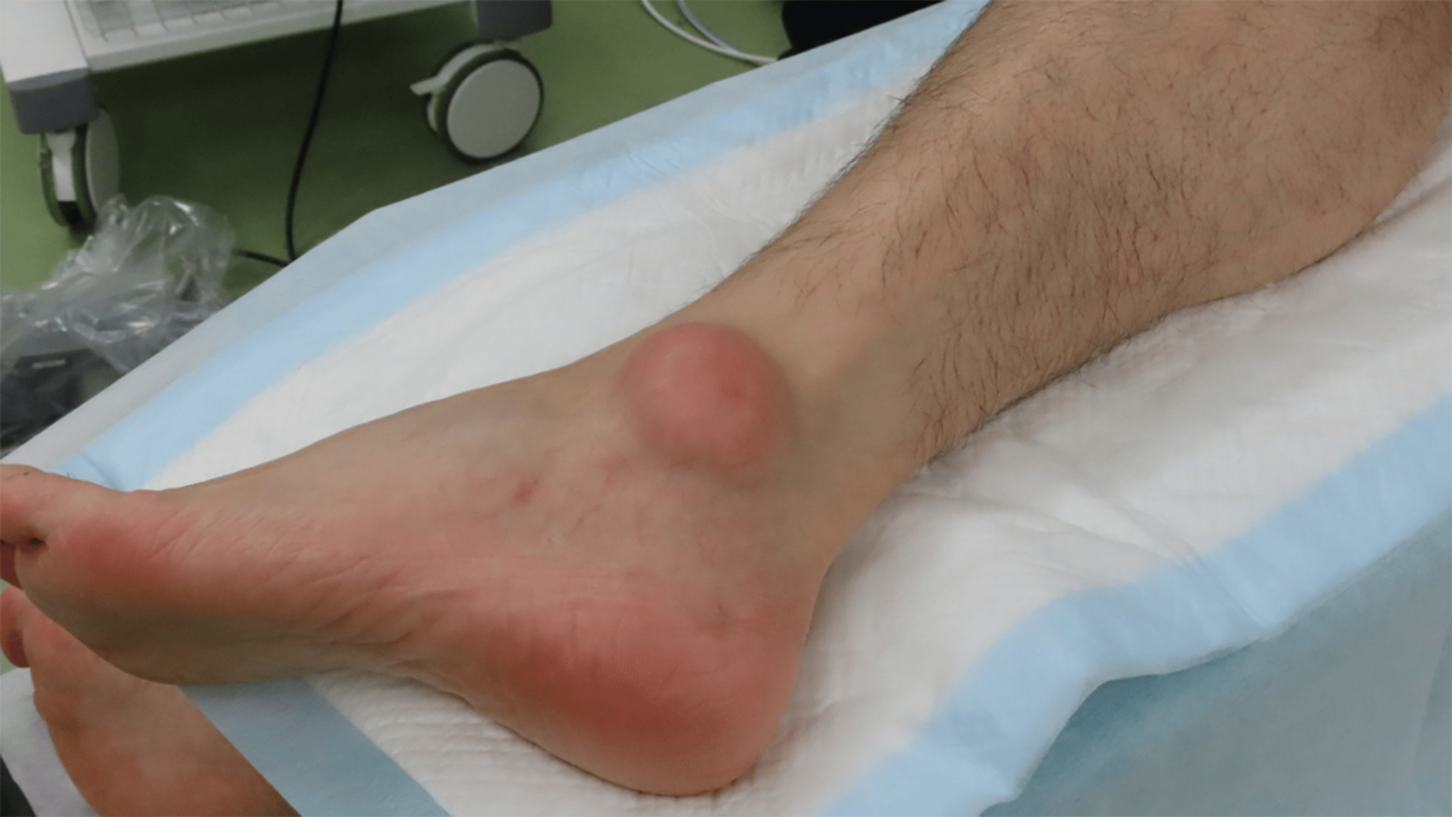
Preoperative clinical appearance Clinical photograph of the left ankle demonstrating a quail egg-sized erythematous soft tissue mass located directly over the lateral malleolus, which caused pain and difficulty with shoe wear.

Endoscopic surgery was performed with the patient in the lateral decubitus position under general anesthesia, using a thigh tourniquet (Figure 4). Radiofrequency devices were not used to avoid thermal skin injury.

**Figure 5:**
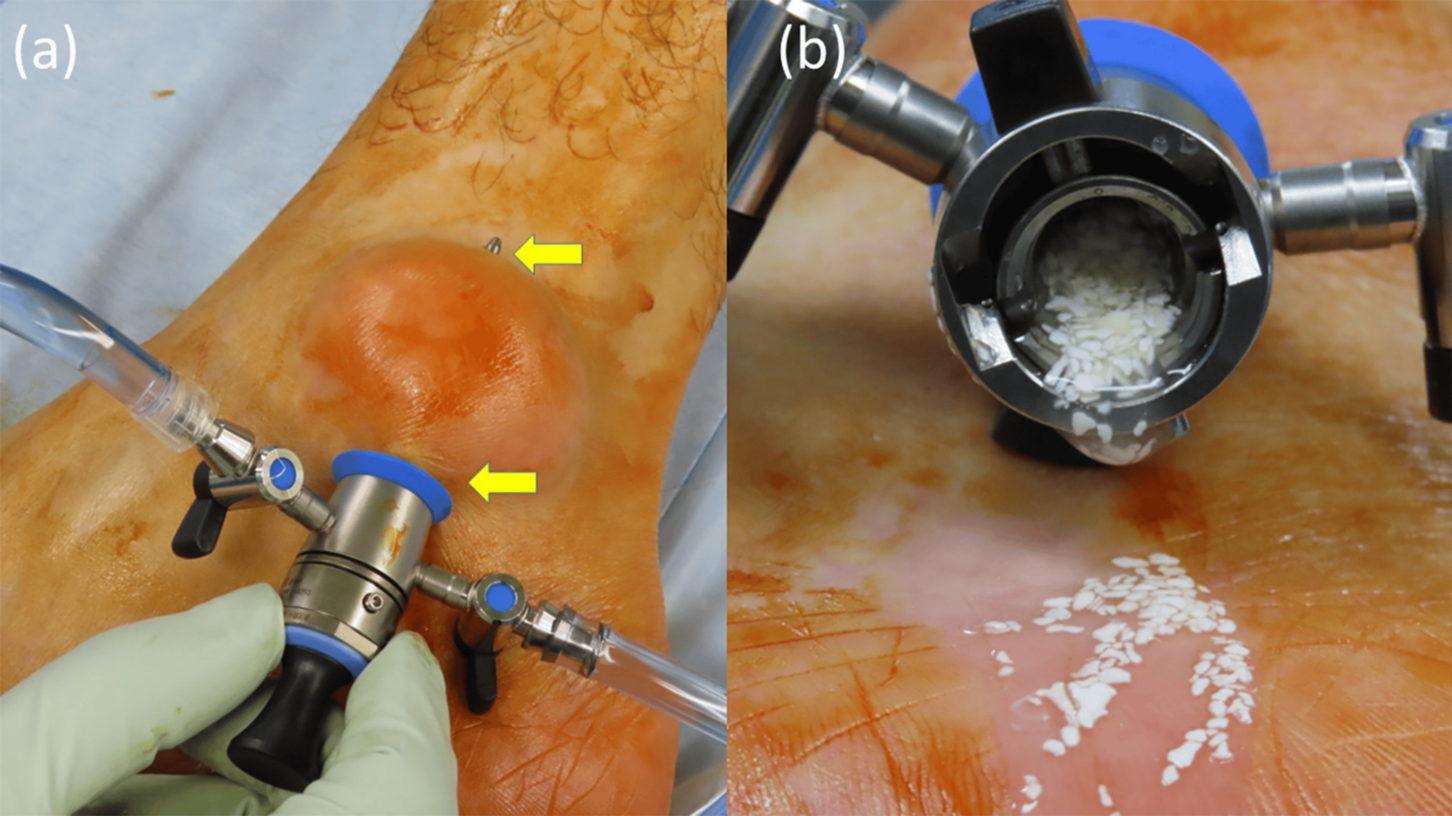
Intraoperative gross appearance (a) Intraoperative photograph showing penetration of the gouty tophus with a trocar to create an endoscopic portal. Creation of proximal and distal portals (arrow) through the tophus, following an extra-articular endoscopic approach similar to that described by Lui [6,7]. (b) Chalky white tophaceous material extruding immediately after removal of the inner sheath following cannula insertion into the tophus.

Two portals were established at the proximal and distal ends of the gouty tophus. A trocar was advanced directly through the tophaceous lesion to create a working tunnel between the portals, following the technique previously described by Lui [6,7] for endoscopic treatment of gouty tophi. Upon insertion of the outer cannula and removal of the trocar, abundant chalky and rice body-like materials were immediately expelled (Figure 5).

**Figure 6:**
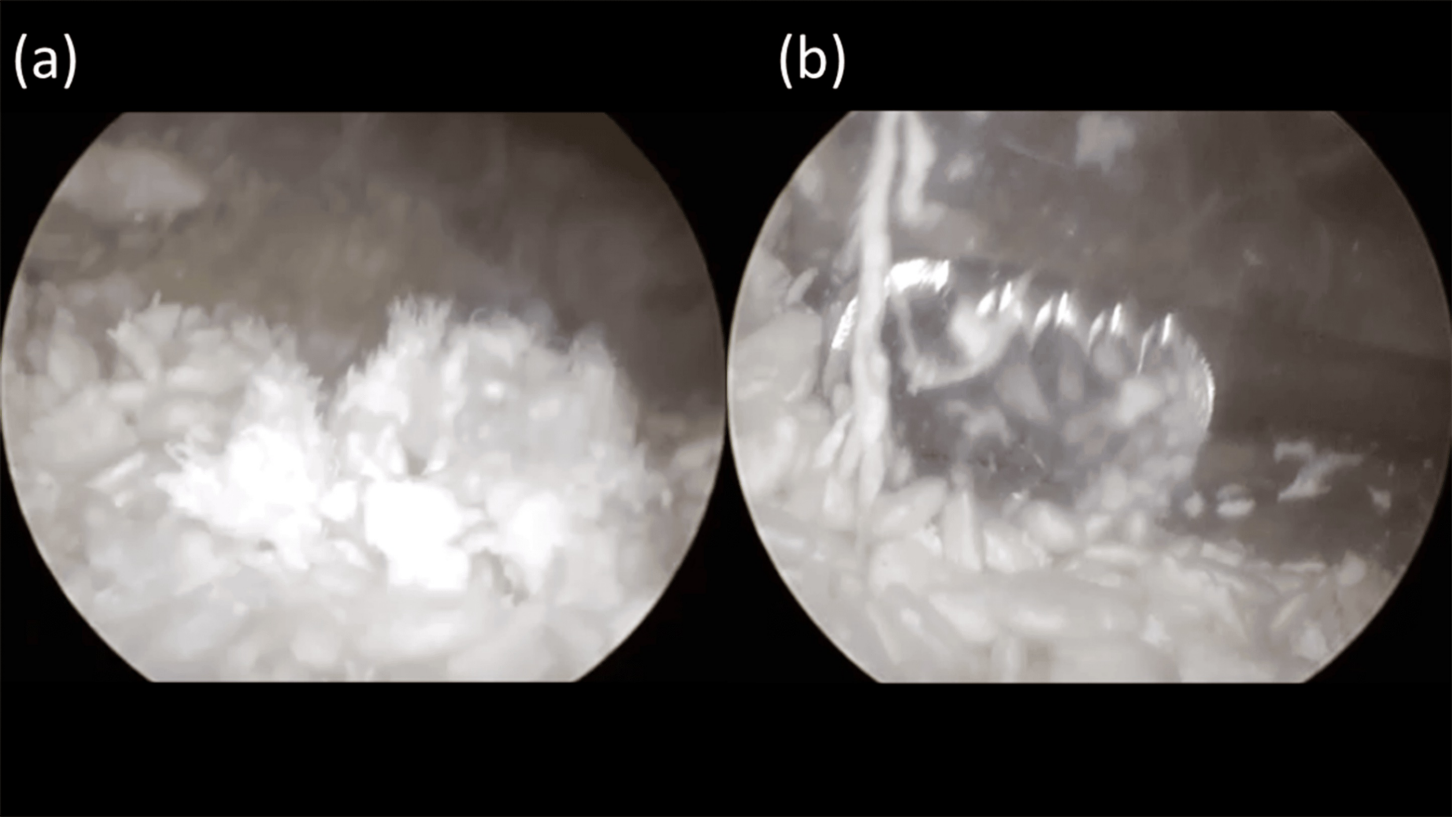
Intraoperative endoscopic findings (a) Endoscopic visualization of abundant chalk-like monosodium urate deposits within the extra-articular cavity. (b) Endoscopic debridement of the tophaceous material using a shaver.

**Figure 7:**
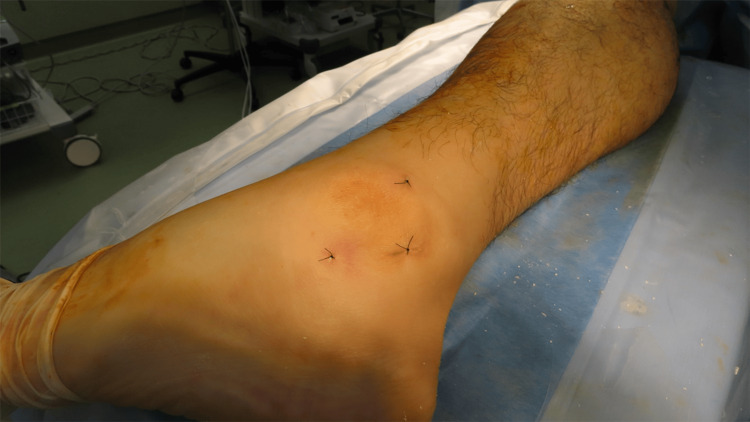
Immediate postoperative appearance Clinical photograph obtained immediately after surgery showing three small portal incisions around the lateral malleolus. Minimal soft tissue disruption is observed, and the skin condition is satisfactory without wound complications.

Under endoscopic visualization, extensive tophaceous deposits and hypertrophic synovium were debrided using a motorized shaver, beginning within the tunnel and extending circumferentially toward the periphery of the lesion (Figure 6). During synovial debridement, focal posterior skin penetration occurred due to dense adhesion between the tophus and overlying skin; this opening was subsequently utilized as a third posterior portal. The defect was managed with local wound care, and no postoperative skin necrosis or infection developed (Figure 7).

Although preoperative imaging suggested intra-articular involvement, no arthroscope was introduced into the ankle joint. The procedure was completed entirely as an extra-articular endoscopic resection. After thorough irrigation, the portals were closed with nylon sutures.

**Figure 8:**
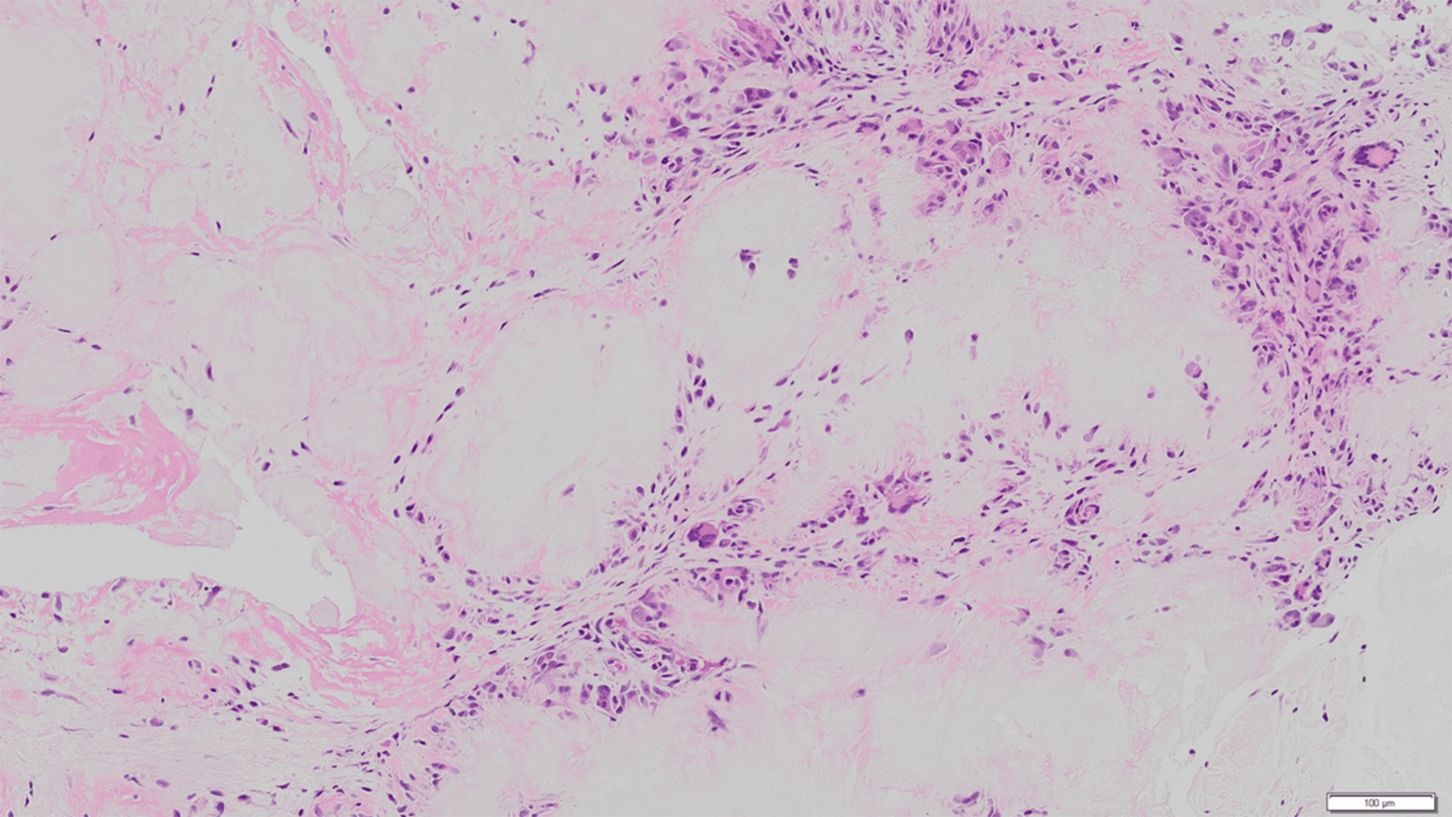
Histopathological findings Histopathological examination with hematoxylin and eosin staining demonstrates amorphous pale eosinophilic deposits representing dissolved monosodium urate crystals, surrounded by a foreign body granulomatous reaction composed of multinucleated giant cells, histiocytes, and lymphocytes, consistent with tophaceous gout.

Histopathological examination of the excised tissue revealed large aggregates of amorphous material consistent with monosodium urate crystal deposition (Figure 8). These deposits were surrounded by a pronounced foreign body granulomatous reaction composed of multinucleated giant cells, histiocytes, and lymphocytes. These findings confirmed the diagnosis of gouty tophus.

Postoperatively, the patient was allowed immediate weight-bearing ambulation using a wooden-based sandal. The wound healed without infection or skin necrosis. Oral febuxostat therapy was initiated for the management of hyperuricemia.

**Figure 9:**
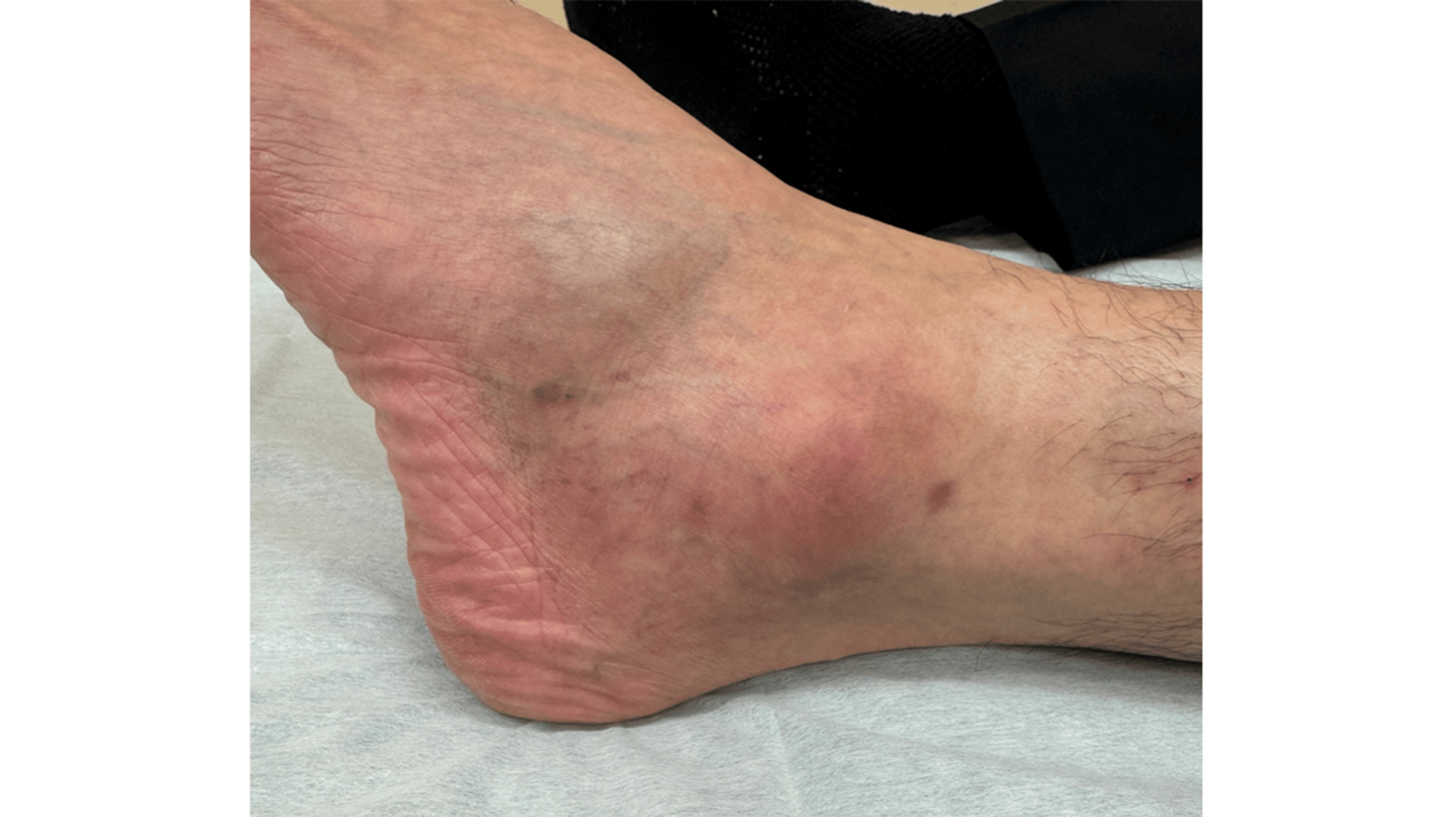
Clinical appearance at six-month follow-up Clinical photograph of the left ankle at six months postoperatively shows marked reduction of the lateral malleolar swelling. Mild residual erythema is present, without evidence of recurrence, infection, or functional limitation.

At six months postoperatively, the lateral malleolar swelling had markedly improved, and the patient was able to wear shoes comfortably. Mild residual redness persisted, but there was no pain, functional limitation, or evidence of recurrence (Figure 9). Postoperative serum uric acid level was 5.8 mg/dL under resumed urate-lowering therapy, achieving the recommended metabolic target (<6 mg/dL). 

## Discussion

Gout is a metabolic disorder caused by deposition of monosodium urate crystals and may progress to chronic tophaceous disease when serum uric acid levels remain uncontrolled [1,2]. Gouty tophi are generally regarded as extra-articular lesions; however, they may extend into adjacent joints, bursae, or tendon sheaths, particularly around the ankle and foot [3-5]. Consequently, distinguishing between intra-articular and extra-articular involvement can be challenging, even with advanced imaging modalities.

In the present case, magnetic resonance imaging demonstrated joint effusion and periarticular inflammatory changes with rice body-like structures, raising suspicion of intra-articular pathology. The differential diagnosis of a chronic lateral malleolar soft tissue mass includes tumorous lesions, synovial chondromatosis, ganglion cyst, and chronic bursitis. In the present case, imaging characteristics, aspiration findings, and histopathology supported the diagnosis of tophaceous gout. The tophus was clearly localized extra-articularly over the lateral malleolus on clinical inspection and computed tomography. Complete symptom relief and sufficient decompression were achieved through extra-articular endoscopic resection alone, without introducing an arthroscope into the ankle joint. This outcome suggests that intra-articular arthroscopy may not always be necessary, even when imaging findings appear equivocal.

Surgical excision of gouty tophi around the lateral malleolus is technically demanding because of thin soft tissue coverage and a high risk of wound complications. Open excision has been associated with delayed wound healing, skin necrosis, and infection in this anatomically vulnerable region. Endoscopic approaches offer the advantage of minimizing skin incision size and soft tissue disruption, potentially reducing these risks [6,7].

The surgical technique employed in this case is conceptually similar to the endoscopic methods reported by Lui for gouty tophi of the first metatarsophalangeal joint and olecranon [6,7]. In those reports, a trocar was advanced directly through the tophaceous lesion to create a working tunnel, enabling effective removal of chalky deposits under endoscopic visualization. Our approach followed the same principle; however, unlike prior technique-focused reports, the present case emphasizes clinical decision-making in a periarticular ankle lesion and demonstrates that satisfactory outcomes can be achieved without routine intra-articular arthroscopy. In this case, intra-articular arthroscopy was avoided based on three factors: (1) absence of bone erosion or joint destruction on CT, (2) preserved ankle range of motion without intra-articular symptoms, and (3) intraoperative confirmation of an encapsulated extra-articular lesion. These criteria may help identify cases suitable for isolated extra-articular endoscopic excision.

Postoperatively, mild residual redness persisted at six months, which may reflect ongoing low-grade inflammatory activity rather than incomplete excision. Importantly, the patient experienced significant functional improvement and was able to wear shoes without difficulty. Long-term urate-lowering therapy remains essential to prevent recurrence, as surgical treatment alone does not address the underlying metabolic disorder [1,2].

Overall, this case highlights the feasibility and effectiveness of extra-articular endoscopic treatment for lateral malleolar gouty tophi and supports its role as a minimally invasive alternative to open excision in selected patients. Chronic tophaceous lesions in areas with thin soft tissue coverage, such as the lateral malleolus, may develop firm adhesion to the overlying skin. Surgeons should be aware of the risk of skin penetration during endoscopic debridement and perform careful blunt dissection in these regions.

Extra-articular endoscopic excision can be an effective and minimally invasive treatment option for selected cases of lateral malleolar tophaceous gout. Careful preoperative imaging assessment and clinical correlation are essential to confirm extra-articular localization and to determine whether ankle arthroscopy can be safely avoided. Awareness of potential skin adhesion in chronic lesions is important to prevent intraoperative complications.

## Conclusions

Extra-articular endoscopic excision is a safe and effective, minimally invasive treatment option for gouty tophi located over the lateral malleolus. Even when periarticular inflammatory changes are suggested by preoperative imaging, adequate decompression and symptom relief may be achieved without routine intra-articular arthroscopy if the primary lesion is clinically extra-articular. This approach minimizes soft tissue damage in an anatomically vulnerable region and may reduce the risk of wound complications associated with open surgery. Careful patient selection combined with appropriate postoperative urate-lowering therapy is essential to achieve favorable functional outcomes and prevent recurrence.
